# Heart Failure in a Patient With Preexisting Giant Hiatal Hernia

**DOI:** 10.7759/cureus.49531

**Published:** 2023-11-27

**Authors:** Satoshi Kurisu, Hitoshi Fujiwara

**Affiliations:** 1 Department of Cardiology, Hiroshima-Nishi Medical Center, Otake, JPN

**Keywords:** computed tomography, ventricular filling, pacemaker, scoliosis, high-flow state

## Abstract

Hiatal hernia is one of common conditions in the elderly. Most patients with hiatal hernia are asymptomatic. However, some reports have described cardiac complications such as heart failure or arrhythmias due to cardiac compression. We report a diagnostically challenging case of heart failure accompanied by preexisting giant hiatal hernia, atrio-ventricular block (AVB)-related bradycardia and aortic valve stenosis (AS). Initial transthoracic echocardiogram (TTE) at the time of 2:1 AVB revealed moderate AS with no cardiac compression by a giant hiatal hernia. Repeated TTE after the correction of heart rate with pacemaker showed decreased peak velocity and mean pressure gradient. The true severity of AS was considered mild under the hemodynamically stable condition. Eventually, AVB-related bradycardia was identified to be responsible for heart failure rather than AS or giant hiatal hernia. The number of diagnostic occasions of heart failure is being increasing especially in the elderly. This case highlights the importance of careful evaluation to determine the major cause of heart failure accompanied by multiple comorbidities.

## Introduction

Hiatal hernia is one of common conditions in the elderly [1,2]. According to the recent study in the general population (aged 53-94 years), hiatal hernia was identified in 9.9% of participants undergoing computed tomography [2]. Most patients with hiatal hernia are asymptomatic; however, the typical symptom is gastroesophageal reflux disease. Less common symptoms are dysphagia, epigastric pain, and chronic iron deficiency anemia [1]. Some reports have described cardiac complications such as heart failure or arrhythmias due to cardiac compression [3,4].

Besides, heart failure may occur in primary myocardial disease and in conditions associated with secondary myocardial dysfunction including bradyarrhythmia or valvular heart disease [5,6]. Heart failure accompanied by multiple comorbidities pose a diagnostic dilemma. 

Here, we report a case of heart failure with preexisting giant hiatal hernia, in which careful evaluation was required to determine its major cause.

## Case presentation

**Figure 1:**
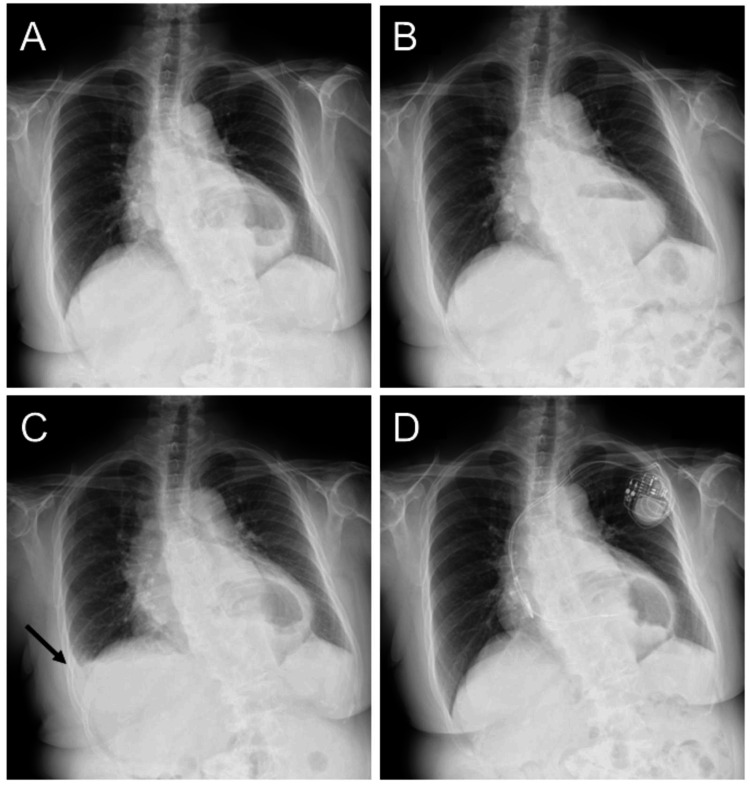
Serial chest radiographs The patient had a history of an asymptomatic hiatal hernia associated with scoliosis. Hiatal hernia had been incidentally found on chest radiographs three years before (A,B). Right-sided pleural effusion newly appeared (arrow), suggesting heart failure (C). Follow-up chest radiograph after pacemaker implantation showed complete resolution of right-sided pleural effusion with similar cardiac silhouette (D).

A 71-year-old woman with rheumatoid arthritis and hypertension, who had been treated with prednisolone (1 mg), methotrexate (2 mg), nifedipine (40 mg), and candesartan (8 mg) once daily in the morning, presented to our hospital with a five-day history of dyspnea. The patient also had a history of an asymptomatic hiatal hernia associated with scoliosis. Hiatal hernia had been incidentally found on chest radiographs three years before (Figure 1).

Her pulse rate was 38 bpm, and blood pressure was 142/80 mmHg. Auscultation found a systolic murmur in the aortic area. Mild peripheral edema was seen in lower extremities, and oxygen saturation was 88%. Oxygen delivery was started via a nasal cannula at a flow rate of 2 L/min. Laboratory studies showed increased brain natriuretic peptide level (Table 1).

**Table 1:** Laboratory data

Variable	Result	Reference range
White blood cell count	3.5 × 10^3^ cells/mm^3^	3.4 - 8.6 × 10^3^ cells/mm^3^
Red blood cell count	2.78 × 10^6^ cells/mm^3^	3.69 - 4.91 × 10^6^ cells/mm^3^
Hemoglobin	9.0 g/dL	11.4 - 15.1 g/dL
Hematocrit	28.6%	34.9 - 45.1%
Platelet count	180 × 10^3^ cells/mm^3^	149 - 351 × 10^3^ cells/mm^3^
Total bilirubin	0.81 mg/dL	0.3 - 1.2 mg/dL
Aspartate aminotransferase	81 U/L	13 - 33 U/L
Alanine aminotransferase	80 U/L	6 - 27 U/L
Creatine phosphokinase	124 U/L	45 - 163 U/L
Creatine phosphokinase-MB	7.3 U/L	0 - 5.7 U/L
Blood urea nitrogen	37.2 mg/dL	8 - 22 mg/dL
Creatinine	0.92 mg/dL	0.40 - 0.79 mg/dL
Brain natriuretic peptide	721.8 pg/mL	< 18.4 pg/mL

**Figure 2:**
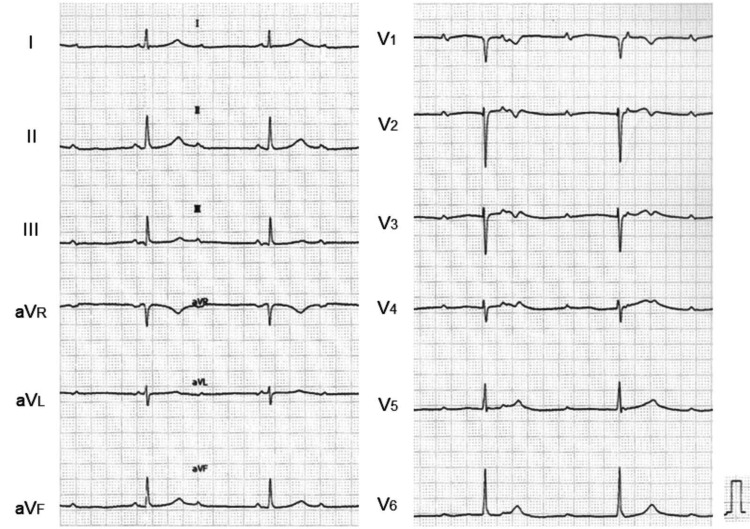
Electrocardiogram An electrocardiogram showed AVB with a heart rate of 36 bpm. AVB: Atrio-ventricular block

A chest radiograph revealed enlarged cardiac silhouette with an abnormal gas inside the mediastinum. In contrast to the constant condition of scoliosis, the size of abnormal gas varied from day to day. Right-sided pleural effusion newly appeared, suggesting heart failure (Figure 1C). An electrocardiogram showed atrio-ventricular block (AVB) with a heart rate of 36 bpm (Figure 2).

**Figure 3:**
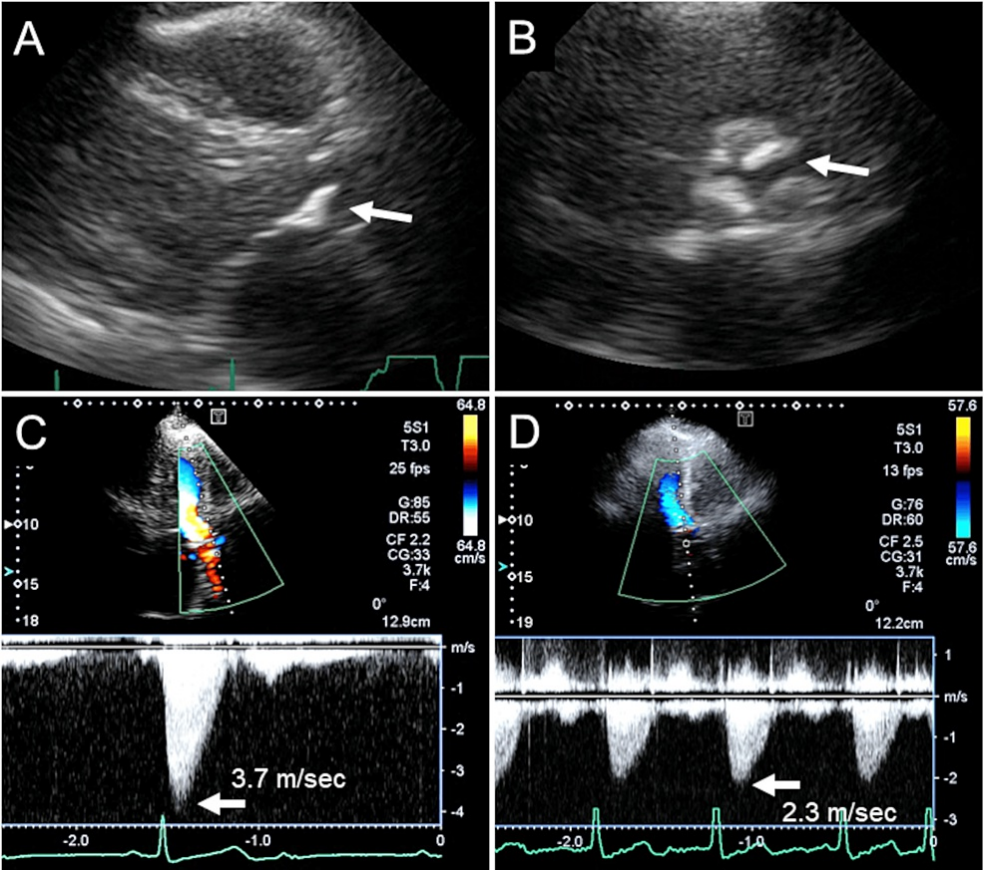
Serial TTEs Initial TTE, which was performed at the time of 2:1 AVB, revealed normal LV systolic function and calcified aortic valve (arrows) (A,B). The severity of AS was considered moderate based on TTE measurements (C). Repeated TTE was performed after the correction of heart rate with pacemaker to evaluate the severity of AS again. The true severity of AS was considered mild under the hemodynamically stable condition (D). TTE: Transthoracic echocardiogram; AVB: Atrio-ventricular block; AS: Aortic valve stenosis; LV: Left ventricular

Initial transthoracic echocardiogram (TTE) (Figure 3), which was performed at the time of 2:1 AVB, revealed normal left ventricular (LV) systolic function and calcified aortic valve (Figure 3A and B). Cardiac compression by a giant hiatal hernia was not detected. The severity of AS was considered moderate based on TTE measurements (Figure 3C, Table 2). She was admitted as an emergency for the treatment of heart failure.

**Table 2:** Serial transthoracic echocardiographic measurements

Parameter	Initial echocardiography	Repeated echocardiography
Heart rate (bpm)	43	84
Left ventricle		
End-diastolic dimension (mm)	47	43
End-diastolic volume (ml)	102	81
End-systolic volume (ml)	23	22
Stroke volume (ml)	79	59
Ejection fraction (%)	78	73
Aortic valve		
Peak velocity (m/sec)	3.7	2.3
Mean pressure gradient (mmHg)	26	12

**Figure 4:**
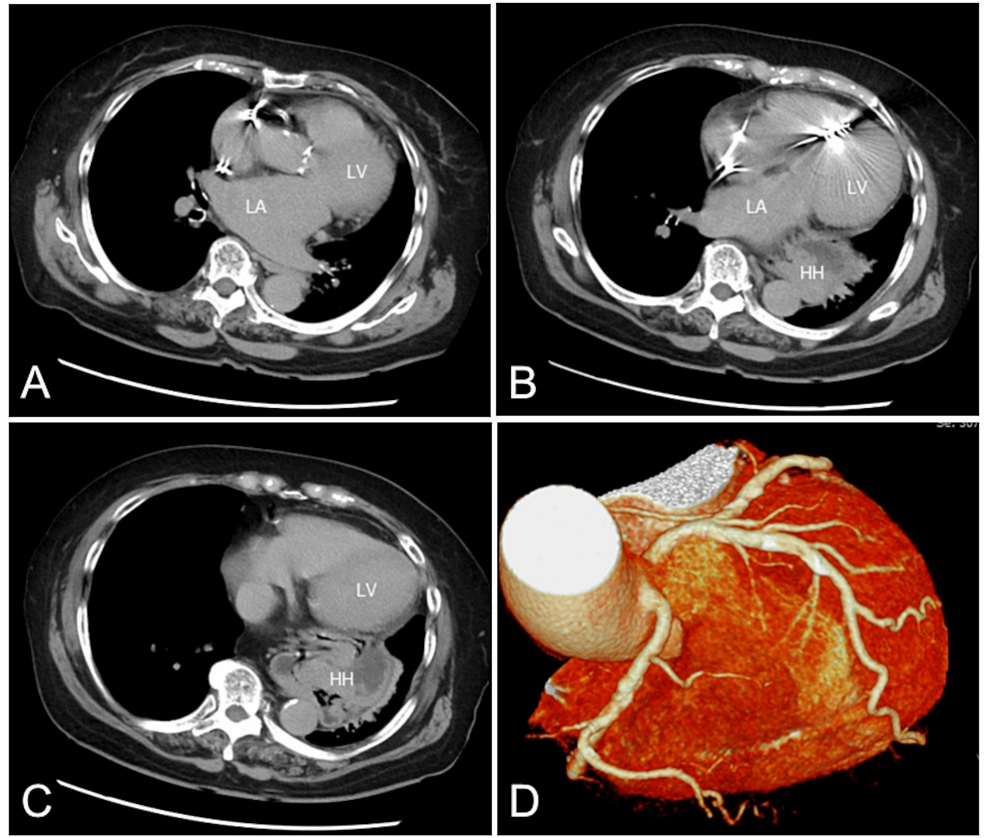
Computed tomographic images Computed tomography revealed calcified aortic valve and a giant hiatal hernia with most of the stomach escaping into the thoracic cavity (A,B,C). No significant coronary atherosclerotic lesions were found (D). The hernia was located behind the LV and LA. There was no significant finding of cardiac compression. LV: Left ventricle; LA: Left atrium

 A dual-chamber pacemaker was implanted in the left pectoral region. On hospital day 5, the patient’s symptom disappeared with no use of diuretics, and oxygen delivery was discontinued. On hospital day 7, follow-up chest radiograph showed complete resolution of right-sided pleural effusion with similar cardiac silhouette (Figure 1D). Computed tomography revealed calcified aortic valve and a giant hiatal hernia with most of the stomach escaping into the thoracic cavity (Figure 4A, B and C). No significant coronary atherosclerotic lesions were found (Figure 4D). The hernia was located behind the LV and left atrium (LA). There was no significant finding of cardiac compression.

Repeated TTE was performed after the correction of heart rate with pacemaker to evaluate the severity of AS again. LV end-diastolic dimension, end-diastolic volume and stroke volume were decreased compared with those on initial TTE (Table 2). Consequently, peak velocity and mean pressure gradient were decreased. The true severity of AS was considered mild under the hemodynamically stable condition (Figure 3D). Eventually, AVB-related bradycardia was identified to be major cause for heart failure rather than AS or giant hiatal hernia. As for AS and giant hiatal hernia, follow-up with watchful monitoring was recommended. She remained in good condition without recurrent heart failure after pacemaker implantation.

## Discussion

In this report, we showed a diagnostically challenging case of heart failure accompanied by preexisting giant hiatal hernia, AVB-related bradycardia and AS. Careful evaluation was required to determine its major cause.

Hiatal hernia is a common condition in the elderly [1,2]. According to the recent study including 3,179 participants undergoing computed tomography, the prevalence of hiatal hernia increased with age, from 2.4% in the sixth decade of life to 7.0%, 14.0% and 16.6% in seventh, eighth and ninth decades, respectively [2]. Risk factors for hiatal hernia include obesity, age-related changes in the diaphragm, and conditions with increased intra-abdominal pressure such as chronic coughing, constipation or scoliosis [7].

A unique aspect of this case was the coexistence of a giant hiatal hernia and heart failure. Although giant hiatal hernias are infrequent, they may lead to rare complications such as heart failure due to cardiac compression [3]. Given the extremely large size of hiatal hernia, we first speculated about the possibility of such condition. However, initial TTE did not detect cardiac compression from a hiatal hernia. Subsequent computed tomography also revealed the same result. Based on the concordant results on two modalities, it was concluded that heart failure occurred independent of the presence of giant hiatal hernia. Next, the patient presented with AVB-related bradycardia in addition to AS. At least in part because bradycardia contributed to the development of heart failure, a dual-chamber pacemaker was implanted [5]. The fact that heart failure improved immediately after pacemaker implantation supported the causal relationship.

The other unique aspect was overestimated severity of AS under the condition of bradycardia [8]. From the viewpoint of LV diastolic filling, 2:1 AVB is different from complete AVB or atrial fibrillation although all can cause bradycardia. Complete AVB includes simultaneous atrio-ventricular contraction on some beats, whereas atrial fibrillation exhibits irregular rhythm beat by beat. These hemodynamic aspects produce variable LV diastolic filling and stroke volume. In contrast, 2:1 AVB exhibits regular rhythm without simultaneous atrio-ventricular contraction; thereby LV diastolic filling and stroke volume are stabilized. Peak velocity and mean pressure gradient are major TTE measurements to determine the severity of AS [9,10]. Because both measurements depend on flow rate across the aortic valve, these values may be falsely increased in certain clinical settings. In the present case, 2:1 AVB was present at the time of initial TTE, which increased diastolic filling and stroke volume, resulting in augmented peak velocity and mean pressure gradient. At the time of repeated TTE after pacemaker implantation, heart rate returned within normal. Consequently, stroke volume, peak velocity and mean pressure gradient were decreased compared with those on TTE. Eventually, the diagnosis of mild AS was made under the hemodynamically stable condition. In the present case, high-flow state induced by bradycardia confounded TTE assessment of AS. It should be recognized that accurate diagnosis of AS requires to confirm the presence or absence of potential factors causing a high-flow state.

## Conclusions

In conclusion, the number of diagnostic occasions of heart failure is being increasing especially in the elderly. This case highlights the importance of careful evaluation to determine the major cause of heart failure accompanied by multiple comorbidities. Making an accurate diagnosis is essential for appropriate treatment of heart failure.
